# The cognitive therapy of depression rests on substantial theoretical, empirical and clinical foundations: a reply to Dr Gipps

**DOI:** 10.1192/pb.bp.116.055616

**Published:** 2017-10

**Authors:** Stirling Moorey

**Affiliations:** 1 South London and Maudsley NHS Foundation Trust, London, UK

## Abstract

Dr Gipps claims that the cognitive therapy for depression rests on a mistake. But his anachronistic analysis of Beck's early research from the perspective of current psychoanalytic theory misses the point. The value of the research was not that it disproved psychoanalytic theory, but that it generated a model of depression that has revolutionised psychotherapy research. Psychoanalysts are belatedly adopting research methods that Beck pioneered half a century ago. The cognitive model of depression has explanatory power for both maintenance and vulnerability and has substantial research underpinning it. Cognitive therapy for depression has a larger body of evidence for its efficacy and relapse prevention effect than any other psychotherapy. Transference-focused approaches to depression have yet to establish themselves in the same way.

Dr Gipps's polemic against Beck's cognitive therapy for depression rests on two assumptions. First, that cognitive therapy ‘doesn't work very well’ for the condition, and second, that the cognitive model is invalid because it is based on a flawed understanding of psychoanalysis. The first statement is misleading – cognitive-behavioural therapy (CBT) has the most comprehensive evidence base of all the psychological therapies. The second statement is largely irrelevant – the cognitive model of depression rests on a substantial body of research that is independent of the experiences that led to its original formulation. This reply will review the evidence for the effectiveness of psychological treatments for depression, highlighting the selectiveness of Dr Gipps's interpretation of the literature. It will then examine the cognitive model, demonstrating that it is far richer than he suggests, and has developed and changed in the light of extensive research. Finally, Gipps's caricature of CBT practice will be challenged: contemporary CBT does have something to say about experiential avoidance and interpersonal processes. It has the flexibility to address these as and when necessary without making them the sole vehicle for change. Gipps seems to believe that by denigrating Beck's psychoanalytic credentials and claiming his theory is based on a misunderstanding he can somehow erase nearly 60 years of research supporting the cognitive model. This is merely opinion, not reasoned scientific discourse.

Dr Gipps argues that current treatments for depression ‘fare little better than placebo’ so we should turn to psychoanalysis. He claims poor results for medication, counselling and CBT, but neglects to mention that trials of psychodynamic psychotherapy have not demonstrated it does better than CBT (the reverse may actually be the case).^1^ If we take a non-partisan approach, the conclusion is that a number of treatments work for depression. For instance, a recent meta-analysis of randomised controlled trials (RCTs) reported effect sizes for CBT, counselling and short-term psychodynamic psychotherapy at 0.67, 0.57 and 0.69, respectively.^2^ Dr Gipps, however, disingenuously selects a small uncontrolled trial of intensive short-term dynamic psychotherapy (*n* = 10) as his argument for the potential superiority of the psychodynamic approach.^3^ All these psychological treatments are as effective as antidepressants, but the superiority of all treatments over placebo may, as he suggests, be less than was once assumed.^4^ There is indeed a powerful placebo effect in mild depression, but this seems to lessen as severity of depression increases.^5–9^ It is also important to note that although placebo may do well in the short term, patients are more likely to relapse.^10^ Cognitive therapy still has the largest evidence base of all the psychotherapies for depression; not only is it effective in the acute phase, but it also reduces relapse.^11,12^ Its effects are not restricted to RCTs: in naturalistic settings 40% of patients recover and 64% show reliable improvement.^13^ The empirical data give us no reason to abandon CBT for depression.

## Psychopathological theory

The implication of Dr Gipps's rather condescending analysis is that cognitive therapy would not have been created if Aaron Beck had been a better psychoanalyst (we are pointedly told that Beck failed entry to the American Institute of Psychoanalysis). According to Gipps, Beck got the wrong end of the stick, and his misunderstanding of theory led him to devise a set of experiments that set psychopathology off on the wrong track for the next half century. The problem here is that Beck's early work is scrutinised from the perspective of current psychoanalytic theory rather than seen in its historical context. It appears the ‘mistake’ Beck made was to be unaware of British psychoanalysis when he carried out his ground-breaking research in the late 1950s. Gipps's main reference is Trevor Lubbe's 2011 book written from the perspective of the British object relations school, which Beck is unlikely to have encountered in his analytic training in the USA – a training inclined towards instinctual and ego theories. Should we then abandon all Freud's work because his theories, originally framed in biological terms, were based on a misunderstanding of modern neurology?^14^ Gipps describes clearly what he sees as the current consensus of psychoanalytic thinking on depression which has unconscious avoidance of emotion at its centre: avoidance of feelings of loss, avoidance of anger towards those to whom we are attached, and avoidance of fear. Rather than face these feelings the patient retreats into depression, preferring to live ‘in a dismal and diminished version of himself, his situation and his future.’ Beck's investigations tested one aspect of the anger-avoidance theory of depression: the masochism hypothesis. Anger towards another is repressed because it is unacceptable, but it breaks through and is then turned on the self. This internalised hostility leads to self-punishment, manifested in the self-criticism and self-abasement found in depression. Gipps wonders why Beck was drawn to the masochism hypothesis to the exclusion of others. The simple answer is that the introjected anger hypothesis was the prevailing theory of depression in American psychoanalysis at the time. Even in 1988 David Milrod could still write that ‘the essential elements in a depression are the self-directed aggression and the mood state to which it leads’.^15^ In this context Beck's investigation of internalised hostility is very reasonable, and his research design perfectly acceptable for its time. But the significance of this research is not that it disproved psychoanalytic theory, but that it stimulated a powerful new theory. Whether one accepts the cognitive model or not, it has made an enormous contribution to the development of empirically based research in depression. Beck showed that it was possible to construct a theory that was refutable, and his steadfast focus on testing his theories has led to changes and modifications on the basis of research evidence. This is in contrast to psychoanalysis, which tends to change on the basis of new theoretical or clinical trends, but rarely in response to new research evidence. The first outcome study of cognitive therapy for depression, for instance, demonstrated that psychological therapy could be as effective as pharmacotherapy and showed that therapy could be operationalised and manualised.^16^ Even psycho-analysts now admit the value of treatment manuals!^17^ Any studies demonstrating the efficacy of psychoanalytic psychotherapy therefore owe a considerable methodological debt to Beck's pioneering work in outcome research.

So what is this mistaken cognitive model of depression? According to Beck, pervasive negative thinking is at the centre of depression: negative views of the self, the world and the future. These thoughts result from an information processing bias that selectively attends to negative events, negatively interprets situations, and encodes them as negative memories. These processes result from the activation of underlying cognitive structures or schemas, in the form of dysfunctional beliefs.^18–20^

There is considerable evidence for this cross-sectional model: for a shift in information processing, for the reciprocal interaction between low mood and negative thinking, and for the presence of dysfunctional beliefs in depression.^21,22^ Beck also formulated a developmental model to explain vulnerability to depression. Negative events in childhood, for example loss of a parent, lead to negative beliefs which become activated when specific events impinge on these schemas. Beck revised this theory in the light of findings that severe life events are not always necessary to precipitate depression. An accumulation of milder stressful events can contribute, and with each successive episode the severity of event needed to trigger depression becomes lower (the kindling effect).^23^ A further refinement of the model came with the discovery that when a depressed mood was evoked people prone to depression exhibited negative cognitive biases (cognitive reactivity).^24^ These new findings were integrated into the theory using the concept of the depressive mode – a network of cognitive affective, motivational, behavioural and physiological schemas activated in depression. The mode is ‘a complex neural network, including multiple relevant brain regions that are activated or deactivated during depression’ (p. 971).^19^ With repeated depressive episodes the network of beliefs becomes stronger and relatively autonomous, so that minor stressors trigger the depressive mode. The cognitive model provides a comprehensive account of both vulnerability to and maintenance of depressive episodes. It does focus on unconscious processes, i.e. schemas and information processing biases, but these are unconscious because they are automatic, not because they are repressed. Regardless of its origins, the cognitive model stands on its own merits as an account of significant phenomena in depression.

## Therapeutic practice

Dr Gipps's main criticism of CBT practice seems to be that it is not psychoanalysis. The collaborative, problem-solving relationship which is at the heart of good CBT is ridiculed as superficial because it apparently misses the opportunity to work with the transference. CBT, unlike psychoanalysis, is a problem-focused therapy, and one of its strengths lies in the way the problem is placed on the table and the therapist and patient work together to solve it. In depression this allows for a partnership in solving realistic problems and the identification of blocks to problem-solving arising from the patient's negative thought processes. For instance, negative predictions about being rejected may lead a patient to be socially avoidant, further reinforcing their depression. The therapist helps the patient test their beliefs by attending a social event and noting the outcome. This ‘collaborative empiricism’, together with the structure and focus of therapy, engages the healthy adult functioning mode and minimises regressive transference so that the patient can learn strategies to help them out of their depression.^25^ When negative interpretations of the therapist's behaviour arise these can be worked on together, for instance by noting how the patient's fear that the therapist may be cross with them is an example of a pattern of depressive misinterpretation that occurs outside the session with others. As the developmental conceptualisation is deepened over the course of therapy, the underlying assumptions that drive this reaction might be identified (e.g. ‘If I make a mistake I'll be rejected’), their origins in parental criticism discussed, and the schemas modified. This is work with the transference but in the service of learning how to manage negative reactions activated when the person is depressed. With patients with personality disturbance the focus on the interpersonal relationship becomes more important and changing interpersonal schemas becomes one of the key goals of therapy This is what Gipps calls the ‘real-time eliciting and challenging of the patient's underlying emotional preoccupations.’ However, CBT has the flexibility to work with the therapeutic process when necessary, but is not shackled to it as the only vehicle for change.

A second, more interesting, point made by Gipps concerns the role of experiential avoidance in depression. This is central to CBT for anxiety, where much of therapy focuses on identifying negative predictions and setting up behavioural experiments to test the fear. This is indeed often done in real time, through experiments in the session such as panic induction. This is of course very effective without any reference to transference. Recent developments in ‘third-wave’ CBT explicitly address emotional avoidance, and there is room for more attention to this within the standard cognitive model of depression. In mindfulness-based cognitive therapy, for instance, mindfulness meditation is taught as a skill to help patients practise moving from the ruminative depressive mode into a mode where they are present for whatever they are experiencing, approaching difficult feelings rather than moving away from them.^26^ Although this concept of avoidance of emotions is similar to that in the psychoanalytic model, the significant differences are that it is not conceptualised as a motivated avoidance arising from a dynamic unconscious, and that it is perfectly possible to work with it outside the transference.

Dr Gipps's final criticism of CBT practice regards its project of ‘dreary self-management’. Psychoanalytic psychotherapy, by contrast, ‘offers an intrinsically mutative emotional exchange which … constitutes a growth in self-possession and a change of heart, obviating the need for such self-management.’ This is wonderful news. Many of us would love to engage in a therapy that magically transforms us so that we do not have to pay attention to our habitual patterns that so frustratingly trip us up, but this is not what good psychodynamic psychotherapy is actually about. It involves noticing reactions outside the session as well as within, and considerable repetitive work on the self. Depression is a relapsing condition and it is patronising and misleading to imply that learning ways to manage it is unnecessary. Patients who successfully negotiate the vicissitudes of recurrent depression learn to identify risk factors that make them vulnerable, recognise how structuring their week helps to maintain positive mood, and how they can easily fall into negative thought patterns. Cognitive therapy gives them tools for doing this, but also changes underlying beliefs, to reduce vulnerability to depression.

## Conclusions

Cognitive therapy is a tried and tested therapy for depression. It has a proven relapse prevention effect, but is not a panacea and is not the only effective treatment for depression. Most of these therapies do not work with the transference, and there is nothing to suggest that a transference-focused therapy will be hugely more effective or reduce dropout rates. The results from the two reported trials of psychoanalytic therapy are promising, but much more research is needed before they can be compared with the accumulated evidence for the effectiveness of CBT. Dr Gipps's article is based on a mistaken reading of the research into CBT as a therapy, and an anachronistic approach which judges Beck's evaluation of psychoanalytic theory in 1959 by the standards of 2016. What Beck developed stands not on this, but on almost 60 years of research. This has not only generated a robust theoretical model of depression, but has given psychoanalysts methodologies they now use to evaluate their own theories and therapies. The cognitive model of depression is likely to be around for another 60 years, but it will evolve and change on the basis of empirical research rather than theoretical whimsy.
